# Supplementary material for: Plasma Polyamine Biomarker Panels: Agmatine in Support of Prostate Cancer Diagnosis
Source: Biomolecules. 2022 Mar 29;12(4):514. doi: 10.3390/biom12040514 (PMC9024899; doi:10.3390/biom12040514)
Supplement: Supplementary file 1 [file biomolecules-12-00514-s001.zip › biomolecules-1596485-supplementary.pdf]

ACETYLPUTRESCINE

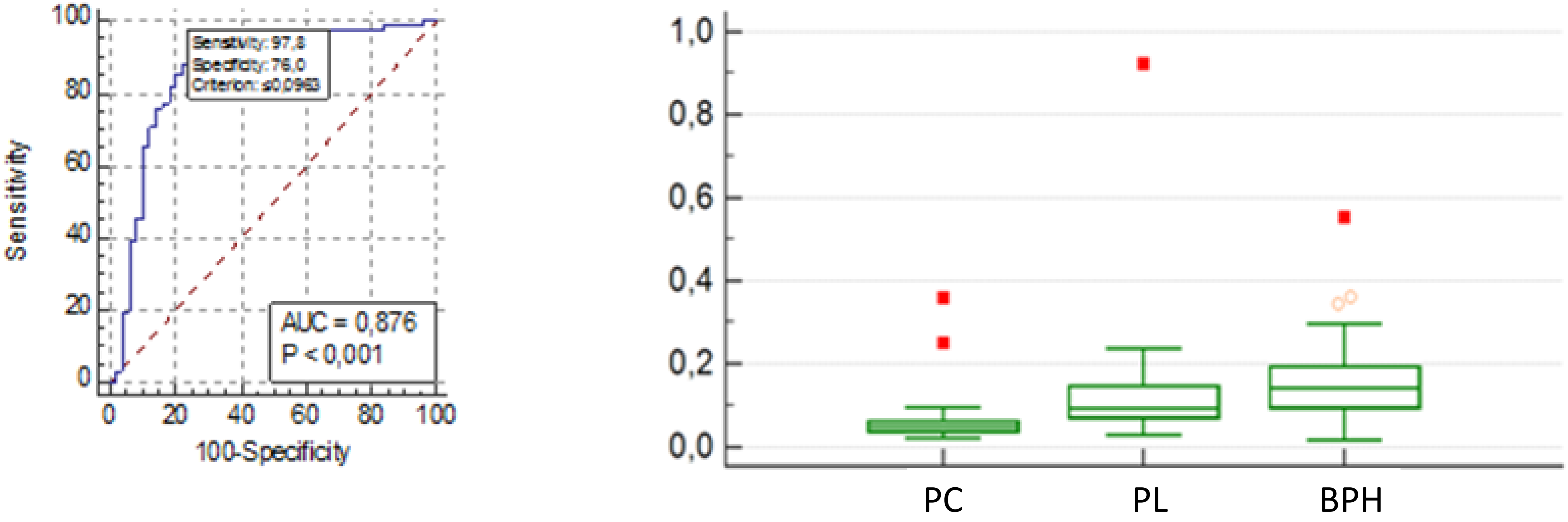

Figure S1. Acetylputrescine ROC curve diagram of Acetylputrescine sensitivity and specificity in prediction of pathological conditions in patients PC and BPH. Boxplots showing the distribution of Acetylputrescine among subjects with prostate cancer (PC, N=92), precancerous lesions (PL, N=26), or benign prostatic hyperplasia (BPH, N=49). The centre line of the boxplots indicates the median (limits of the box indicate the 25th and 75th percentile). The whiskers represent either 1.5 times the interquartile range (IQR) or the maximum/minimum data point if they are within 1.5 times the IQR. Wilcoxon’s test was used to compare mean Agmatine levels among groups.

ACETYLSPERMIDINE

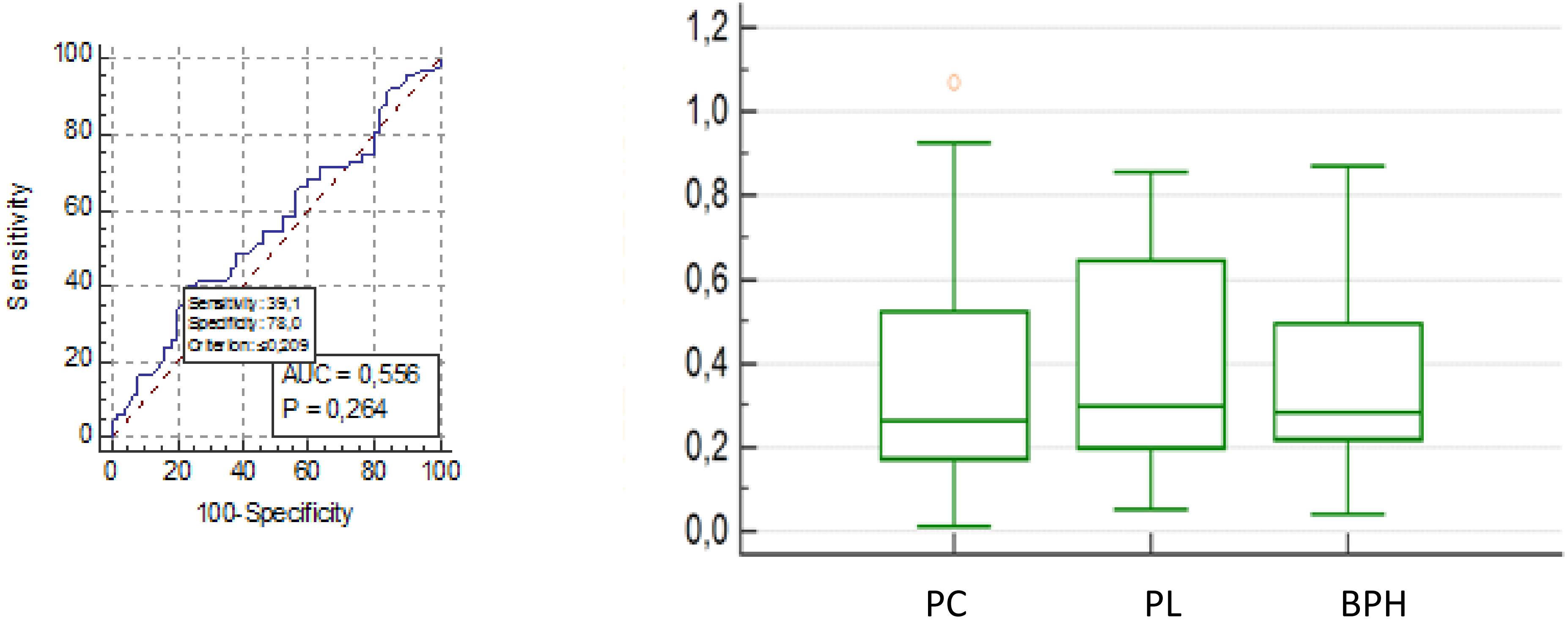

Figure S2. Acetylspermidine ROC curve diagram of Acetylspermidine sensitivity and specificity in prediction of pathological conditions in patients PC and BPH. Boxplots showing the distribution of Acetylspermidine among subjects with prostate cancer (PC, N=92), precancerous lesions (PL, N=26), or benign prostatic hyperplasia (BPH, N=49). The centre line of the boxplots indicates the median (limits of the box indicate the 25th and 75th percentile). The whiskers represent either 1.5 times the interquartile range (IQR) or the maximum/minimum data point if they are within 1.5 times the IQR. Wilcoxon's test was used to compare mean Agmatine levels among groups.

ACETYLSPERMINE

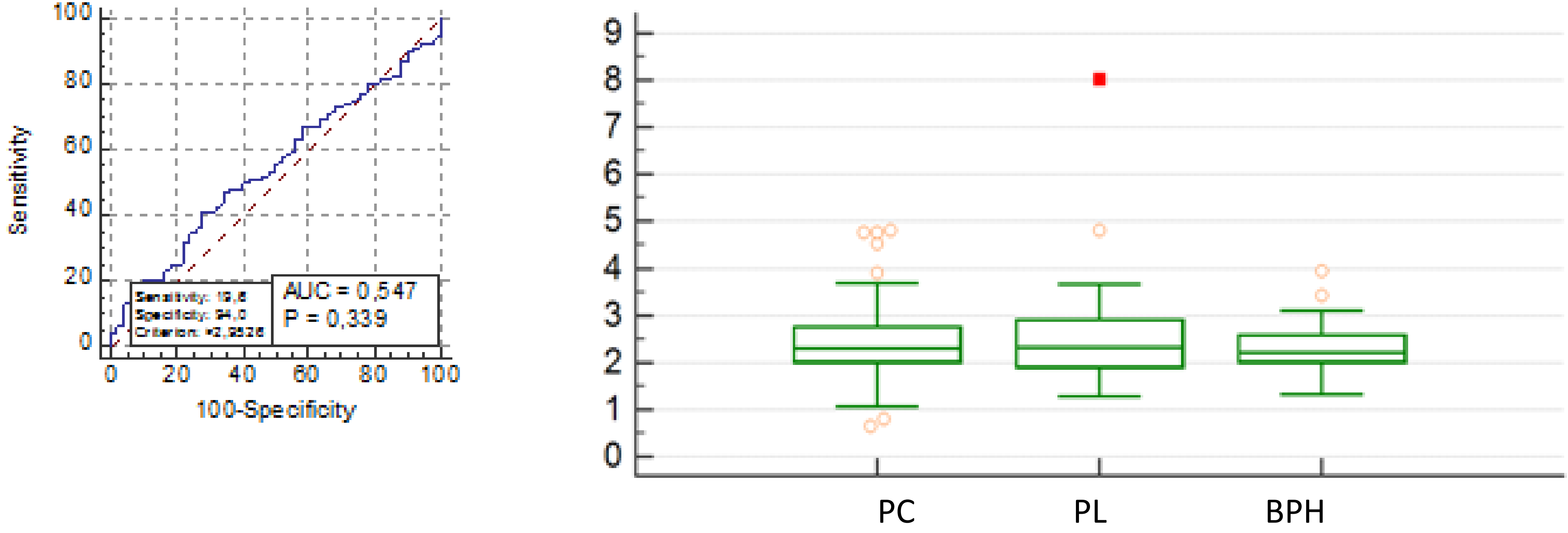

Figure S3. Acetylspermine ROC curve diagram of Acetylspermidine sensitivity and specificity in prediction of pathological conditions in patients PC and BPH. Boxplots showing the distribution of Acetylspermine among subjects with prostate cancer (PC, N=92), precancerous lesions (PL, N=26), or benign prostatic hyperplasia (BPH, N=49). The centre line of the boxplots indicates the median (limits of the box indicate the 25th and 75th percentile). The whiskers represent either 1.5 times the interquartile range (IQR) or the maximum/minimum data point if they are within 1.5 times the IQR. Wilcoxon’s test was used to compare mean Agmatine levels among groups.

CADAVERINE

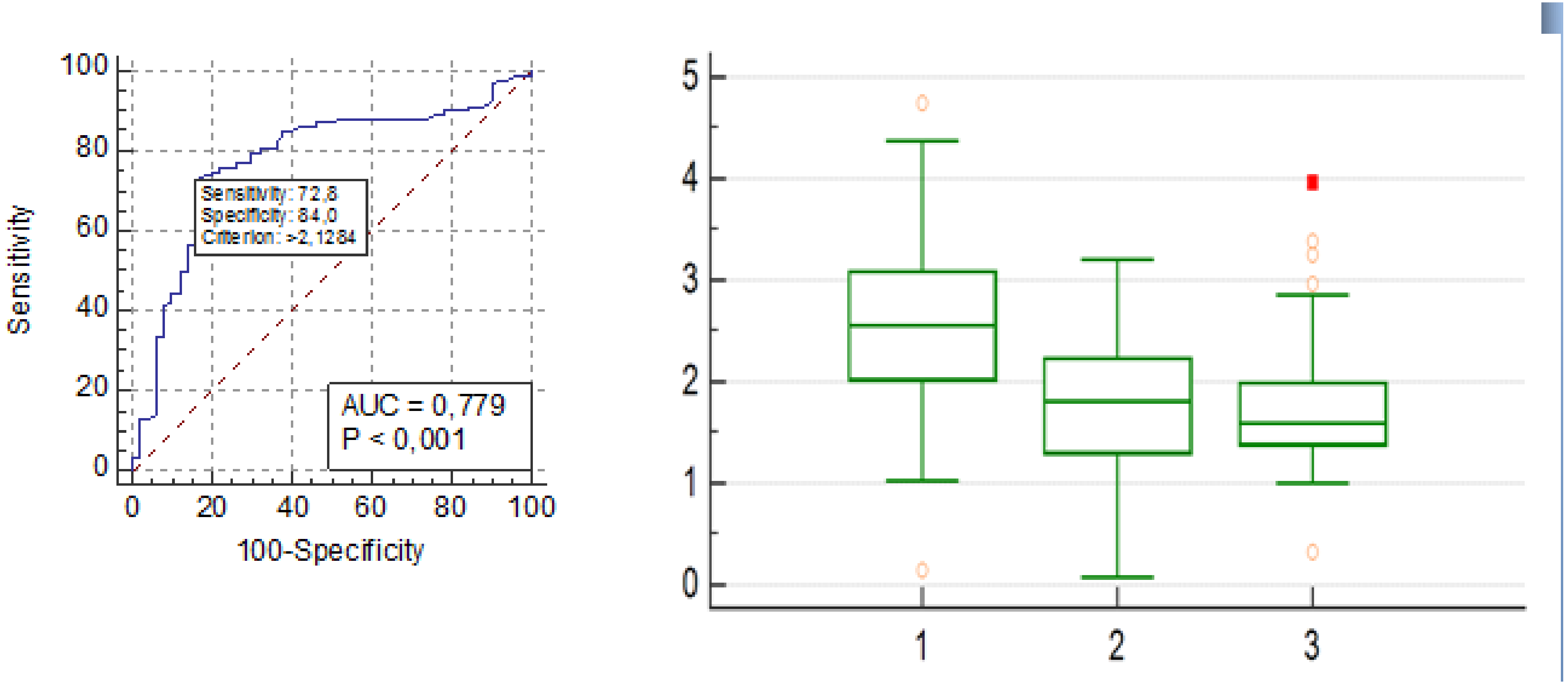

Figure S4. Cadaverine. ROC curve diagram of Cadaverine sensitivity and specificity in prediction of pathological conditions in patients PC and BPH. Boxplots showing the distribution of Cadaverine among subjects with prostate cancer (PC, N=92), precancerous lesions (PL, N=26), or benign prostatic hyperplasia (BPH, N=49). The centre line of the boxplots indicates the median (limits of the box indicate the 25th and 75th percentile). The whiskers represent either 1.5 times the interquartile range (IQR) or the maximum/minimum data point if they are within 1.5 times the IQR. Wilcoxon's test was used to compare mean Agmatine levels among groups.

LYSINE

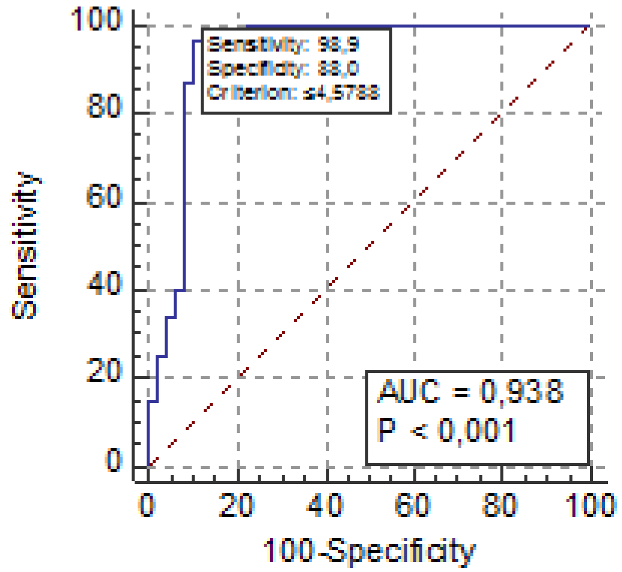

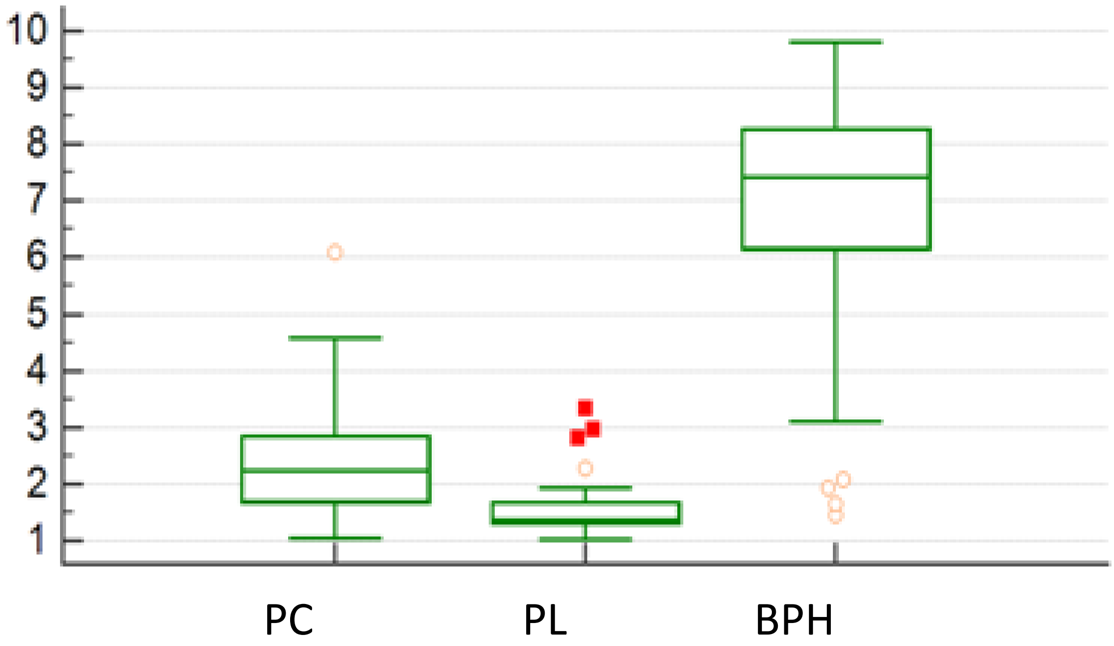

Figure S5. Lysine. ROC curve diagram of Lysine sensitivity and specificity in prediction of pathological conditions in patients PC and BPH. Boxplots showing the distribution of Lysine among subjects with prostate cancer (PC, N=92), precancerous lesions (PL, N=26), or benign prostatic hyperplasia (BPH, N=49). The center line of the boxplots indicates the median (limits of the box indicate the 25th and 75th percentile). The whiskers represent either 1.5 times the interquartile range (IQR) or the maximum/minimum data point if they are within 1.5 times the IQR. Wilcoxon's test was used to compare mean Agmatine levels among groups.

ORNITHINE

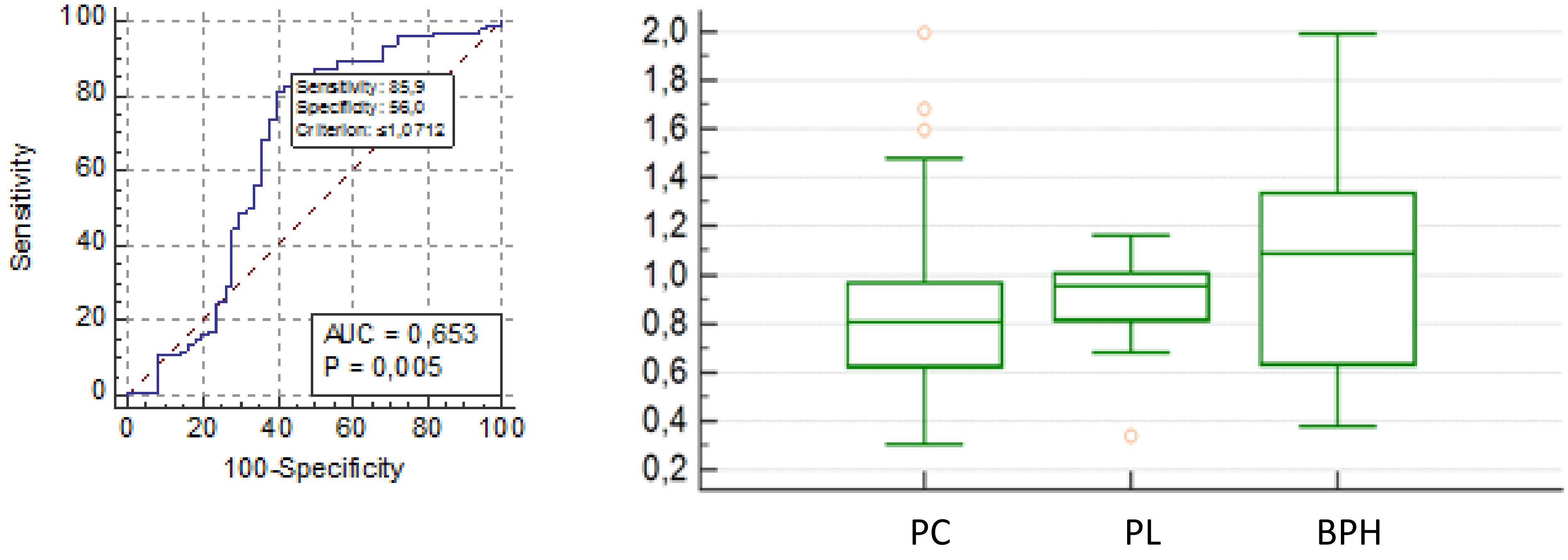

Figure S6. Ornithine. ROC curve diagram of Ornithine sensitivity and specificity in prediction of pathological conditions in patients PC and BPH. Boxplots showing the distribution of Ornithine among subjects with prostate cancer (PC, N=92), precancerous lesions (PL, N=26), or benign prostatic hyperplasia (BPH, N=49). The center line of the boxplots indicates the median (limits of the box indicate the 25th and 75th percentile). The whiskers represent either 1.5 times the interquartile range (IQR) or the maximum/minimum data point if they are within 1.5 times the IQR. Wilcoxon's test was used to compare mean Agmatine levels among groups.

PUTRESCINE

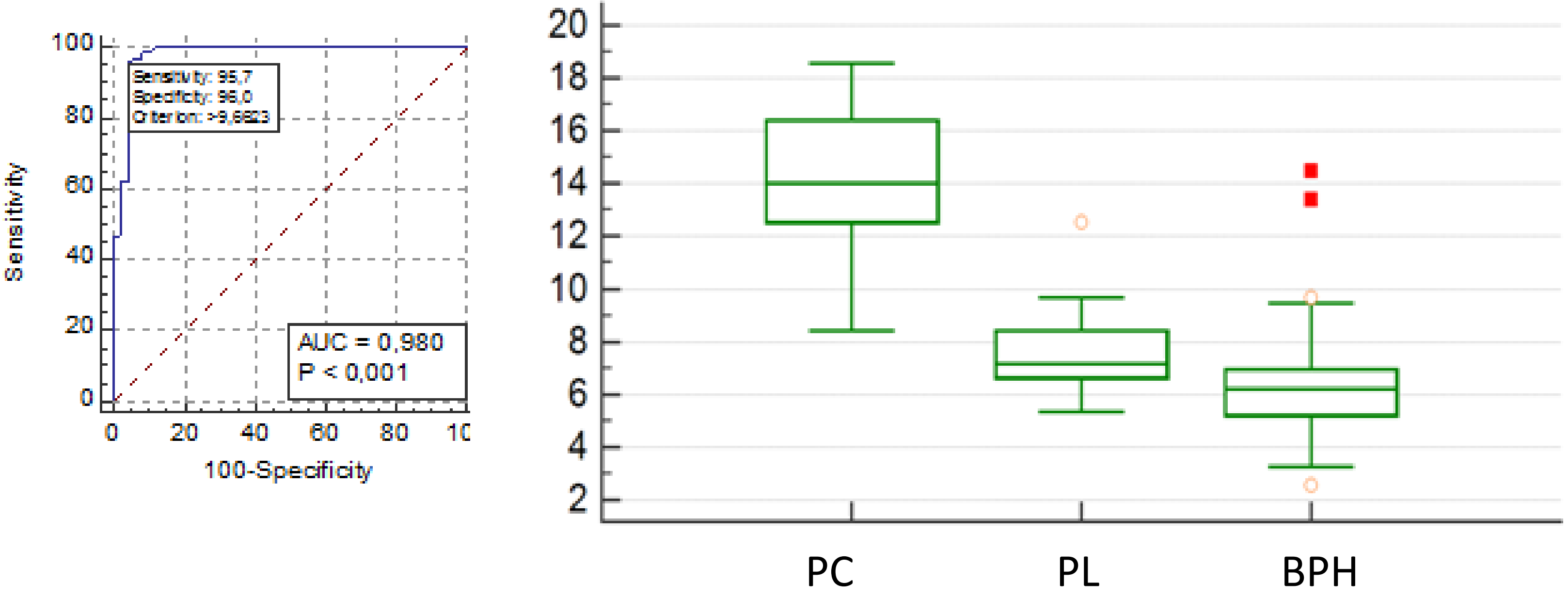

Figure S7. Putrescine. ROC curve diagram of Putrescine sensitivity and specificity in prediction of pathological conditions in patients PC and BPH. Boxplots showing the distribution of Putrescine among subjects with prostate cancer (PC, N=92), precancerous lesions (PL, N=26), or benign prostatic hyperplasia (BPH, N=49). The center line of the boxplots indicates the median (limits of the box indicate the 25th and 75th percentile). The whiskers represent either 1.5 times the interquartile range (IQR) or the maximum/minimum data point if they are within 1.5 times the IQR. Wilcoxon's test was used to compare mean Agmatine levels among groups.

SPERMIDINE

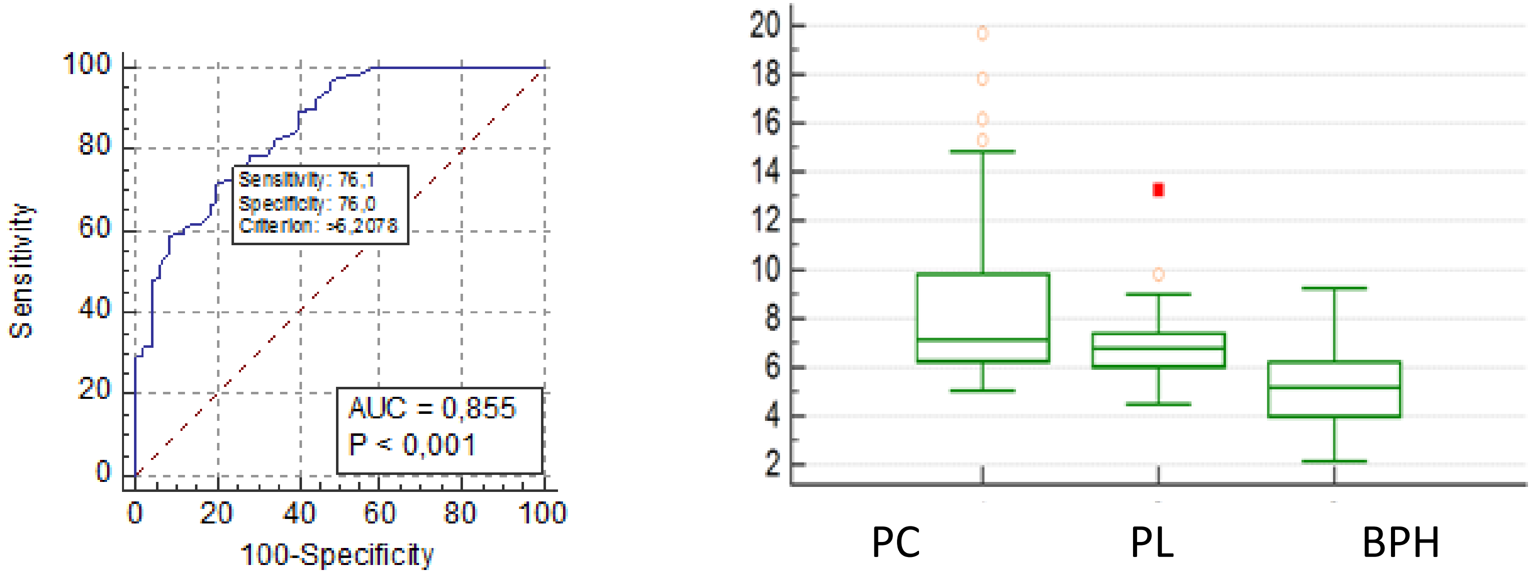

Figure S8. Spermidine. ROC curve diagram of Spermidine sensitivity and specificity in prediction of pathological conditions in patients PC and BPH. Boxplots showing the distribution of Spermidine among subjects with prostate cancer (PC, N=92), precancerous lesions (PL, N=26), or benign prostatic hyperplasia (BPH, N=49). The center line of the boxplots indicates the median (limits of the box indicate the 25th and 75th percentile). The whiskers represent either 1.5 times the interquartile range (IQR) or the maximum/minimum data point if they are within 1.5 times the IQR. Wilcoxon's test was used to compare mean Agmatine levels among groups.

SPERMINE

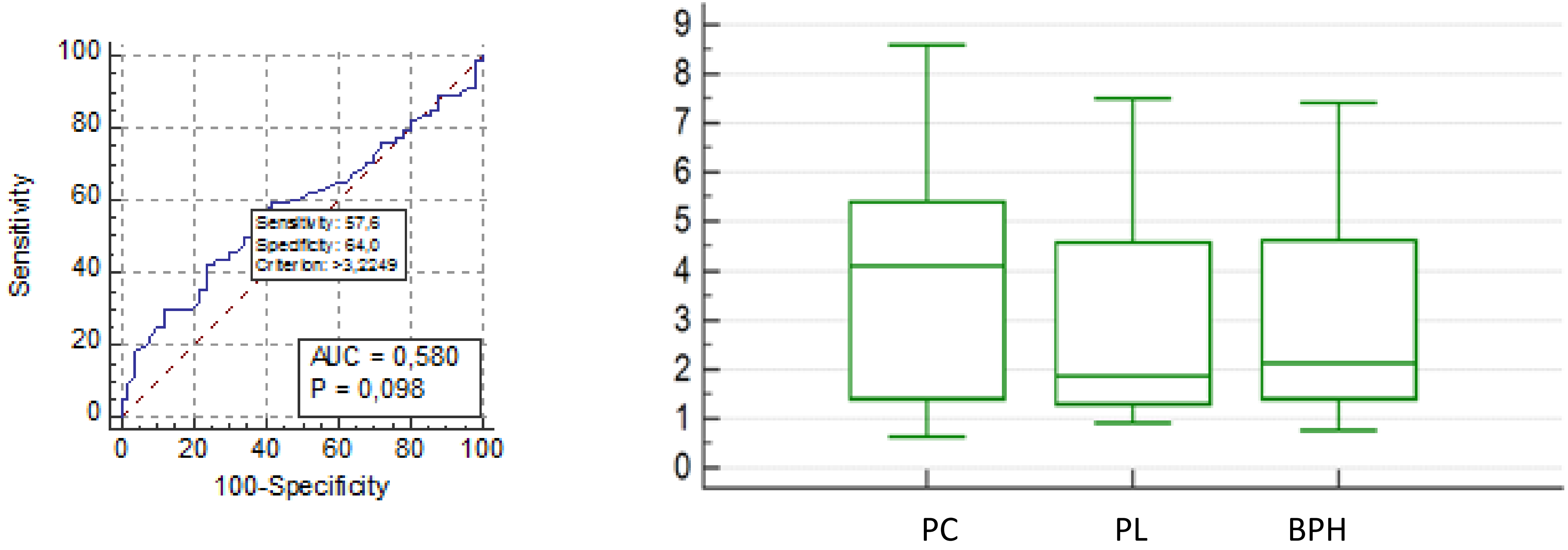

Figure S9. Spermine. ROC curve diagram of Spermine sensitivity and specificity in prediction of pathological conditions in patients PC and BPH. Boxplots showing the distribution of Spermine among subjects with prostate cancer (PC, N=92), precancerous lesions (PL, N=26), or benign prostatic hyperplasia (BPH, N=49). The center line of the boxplots indicates the median (limits of the box indicate the 25th and 75th percentile). The whiskers represent either 1.5 times the interquartile range (IQR) or the maximum/minimum data point if they are within 1.5 times the IQR. Wilcoxon's test was used to compare mean Agmatine levels among groups.

PSA

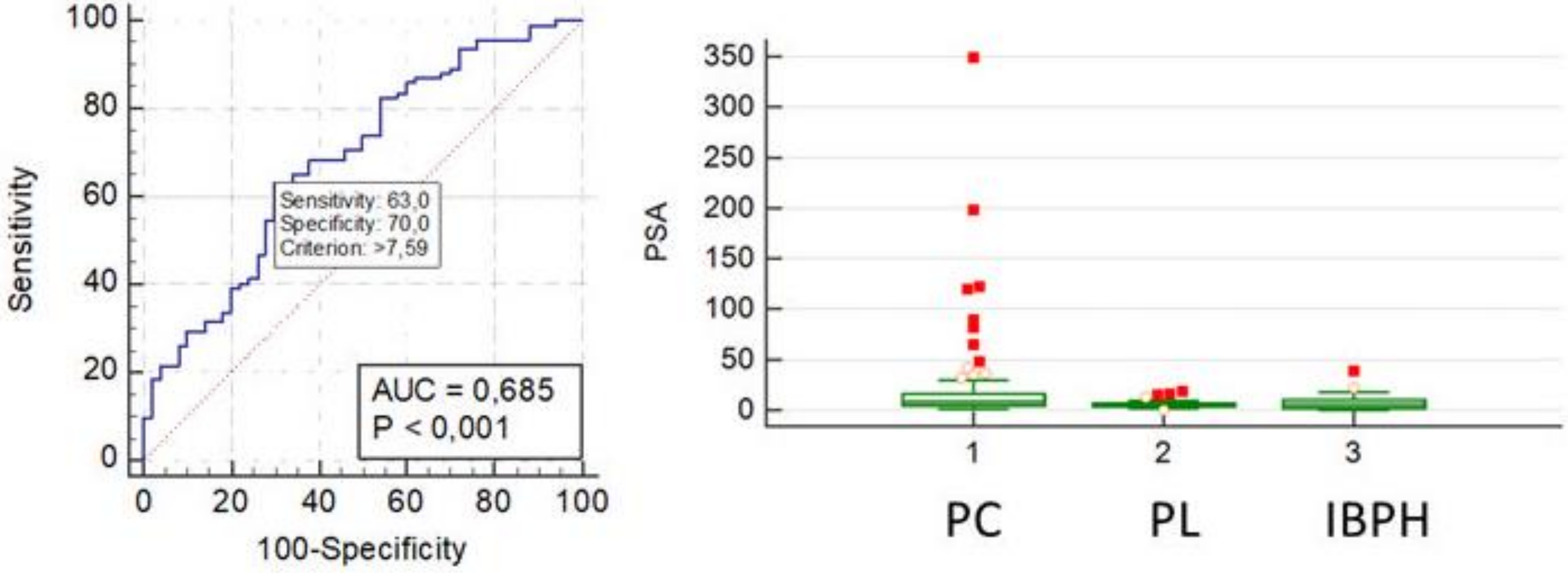

Figure S10. PSA. ROC curve diagram of PSA sensitivity and specificity in prediction of pathological conditions in patients PC and BPH. Boxplots showing the distribution of PSA among subjects with prostate cancer (PC, N=92), precancerous lesions (PL, N=26), or benign prostatic hyperplasia (BPH, N=49). The center line of the boxplots indicates the median (limits of the box indicate the 25th and 75th percentile). The whiskers represent either 1.5 times the interquartile range (IQR) or the maximum/minimum data point if they are within 1.5 times the IQR. Wilcoxon's test was used to compare mean Agmatine levels among groups.
